# A woman with a rare p.Glu74Gly transthyretin mutation presenting exclusively with a rapidly progressive neuropathy: a case report

**DOI:** 10.1186/1752-1947-8-403

**Published:** 2014-12-04

**Authors:** Anne Schänzer, Christoph Kimmich, Christoph Röcken, Thomas Haverkamp, Isabell Weidner, Till Acker, Heidrun H Krämer

**Affiliations:** 1 Institute of Neuropathology, Justus Liebig University, Arndstrasse 16, 35392 Giessen, Germany; 2 Medical Department V, Amyloidosis Center, University of Heidelberg, Im Neuenheimer Feld 410, 69120 Heidelberg, Germany; 3 Institute of Pathology, Christian-Albrechts-University, Arnold-Heller-Strasse 3, 24105 Kiel, Germany; 4 MVZ Dr Eberhard & Partner Dortmund, Laboratory Medicine Dortmund, Brauhausstrasse 4, 44137 Dortmund, Germany; 5 Department of Neurology, Justus Liebig University, Klinikstrasse 33, 35385 Giessen, Germany

## Abstract

**Introduction:**

Familial amyloid polyneuropathy is a rare autosomal dominant disorder caused by mutations in the transthyretin gene, *TTR*. Diagnosis can be challenging, especially if other family members are not affected or an obvious systemic involvement is lacking. The patients are often misdiagnosed, leading to a delay in the initiation of therapy.

**Case presentation:**

A 35-year-old woman of Turkish origin presented to our outpatient clinic with severe polyneuropathy associated with distally pronounced tetraparesis and hypesthesia of 2 to 3 years’ duration. In addition, small nerve fiber involvement with impaired detection of cold temperatures and tingling pain in the lower legs was reported. She did not complain of autonomic dysfunction or visual disturbance. Her family history was empty regarding neuromuscular disorders. The routine diagnostic work-up was unremarkable. A sural nerve biopsy disclosed amyloid deposits, which led to the identification of a rare heterozygous transthyretin mutation (p.Glu74Gly; old classification: p.Glu54Gly).

**Conclusions:**

Few cases with this very heterozygous mutation can be found in the literature. In contrast to the case of our patient, all of the previously described patients in the literature presented with additional severe autonomic symptoms, involvement of the eyes and a positive family history. In this case report, we emphasize that, in patients with progressive neuropathy with small fiber involvement, an amyloid neuropathy should be considered in the differential diagnosis, even if the family history is empty and other organs are not affected.

## Introduction

Amyloid polyneuropathies are characterized by pathological deposits of misfolded proteins and peptides in a β-pleated sheet conformation, which induce damage and a subsequent loss of large- as well as small-diameter nerve fibers. Familial amyloidoses are rare diseases, especially in nonendemic areas, and are divided into three subtypes that present with different involvement of the peripheral nervous system: transthyretin (TTR), apolipoprotein A-I and gelsolin. TTR-associated familial amyloid polyneuropathy (TTR-FAP) is the most common type, in which amyloid depositions in peripheral nerves lead to a progressive sensorimotor and autonomic neuropathy. TTR-FAPs are autosomal dominant disorders with a variable age of onset between 30 and 70 years, as well as an early (<50 years) and late (>50 years) onset of presentation. A rapidly progressive polyneuropathy is described in most cases. More than 100 mutations in the *TTR* gene are known, with Val50Met (p.Val30Met) being the most common one. The clinical manifestation can be very heterogeneous; thus, the diagnosis is difficult and might be delayed, especially if the patient is young and the family history negative [1–7]*.* Early diagnosis is important because treatment options are available. In this report, we describe the clinical, electrophysiological, histopathological and genetic findings in a Turkish woman with a rapidly progressive type of TTR-FAP. To the best of our knowledge, this report is the first in the literature describing a patient who presented exclusively with severe neuropathy as a symptom of the rare p.Glu74Gly mutation.

## Case presentation

A 35-year-old woman of Turkish origin presented to our outpatient clinic with increasing gait and stance disturbances of 2 or 3 years’ duration. She reported tingling pain in the feet and lower legs as well as an impairment in detecting cold temperatures as a sign of small fiber involvement. In addition, the patient had recurrent shortness of breath. She had previously been diagnosed with a severe polyneuropathy of unknown origin. Besides the neuropathy, her medical history was empty. She denied gastrointestinal or visual disturbances.

She was a daughter of nonconsanguineous parents from Turkey. Her family history was empty regarding neuromuscular disorders. She has four brothers and two sisters. She is the mother of two healthy children (ages 5 and 7 years).

Her clinical examination revealed a distal as well as left pronounced tetraparesis, hyporeflexia with absence of Achilles tendon reflexes, hypesthesia of the lower legs reaching the patellae and pallanesthesia at the medial malleoli. No compound motor unit potential could be measured on her lower extremities of the tibial or peroneal nerves bilaterally. Motor conduction studies of her upper extremities, including investigation of both the median and ulnar nerves, were unremarkable. No sensory nerve action potentials could be obtained on the upper or lower extremities. Signs of denervation in the distal muscles of the lower extremities, as well as signs of chronic neurogenic reorganization, were detected by electromyography. The extensive polyneuropathy work-up and rheumatology assessments did not produce any pathological results. No indication for a paraneoplastic origin of the disease was discovered. Standard screening methods did not reveal an associated monoclonal gammopathy of undetermined significance or diabetes. However, proteinuria was discovered in the 24-hour urine collection, indicating renal involvement. Because she had dyspnea, a cardiac work-up was performed. In this clinical examination, minor signs of chronic heart failure were found, and we classified her as New York Heart Association functional class II. Magnetic resonance imaging of her heart revealed early left ventricular hypertrophy with a normal ejection fraction of 60%. Echocardiography showed an increased thickness of the interventricular septum of 15mm and a slightly impaired longitudinal left ventricular function. The general weakness might also have been associated with an involvement of the autonomic nervous system with low systolic blood pressure (generally 90mmHg or lower). The patient did not complain about visual disturbances. However, upon examination, opacity of the vitreous body with signs of visual loss was detected in both eyes.

**Figure 1:**
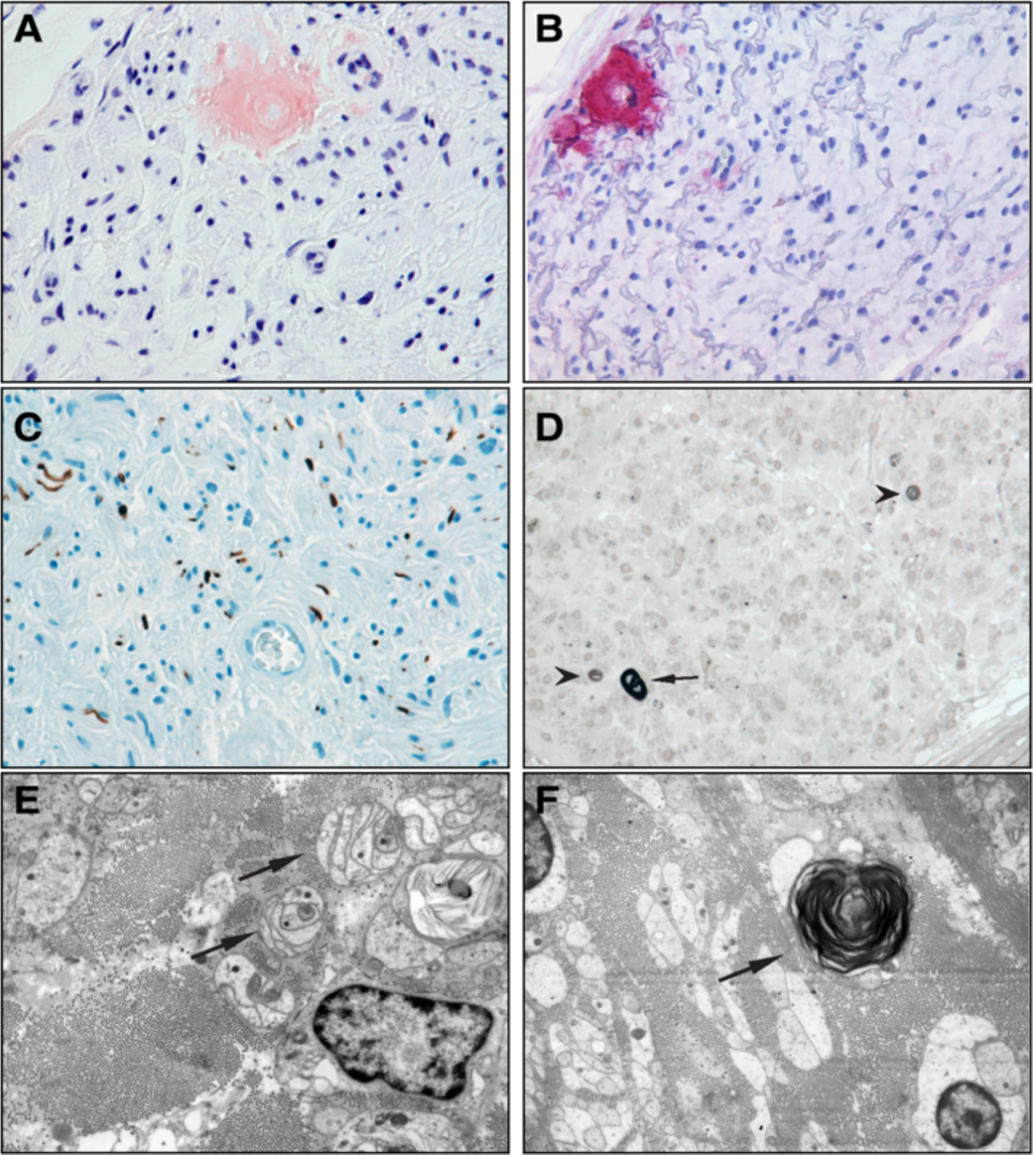
**Sural nerve biopsy sections.** Small endoneural amyloid deposits are shown in the Congo red staining **(A)** with a positive transthyretin antibody reaction **(B)**. There is a severe axonal loss with antibodies against neurofilament **(C)**. In the *p*-phenylenediamine-stained semithin sections, only a few remaining myelinated axons (arrow) can be seen, and acute myelin degeneration (arrowhead) is present **(D)**. Electron microscopic images show denervated Schwann cell bands (arrows) **(E)** and axonal degeneration with myelin debris (arrow) **(F)**.

A sural nerve biopsy was performed and processed using standard protocols [8]. Paraffin-embedded nerve tissue was processed for Congo red staining. The stain depicted small but distinct endoneural deposits of amyloid (Figure 1A) with a bright green birefringence under polarizing filters (data not shown). These deposits stained positive with antibodies against transthyretin (TTR) (Figure 1B). Elastica van Gieson staining allowed visualization of collagen fibers and showed a strong endoneural fibrosis (data not shown). Immunohistochemistry against neurofilament and *p*-phenylenediamine-stained semithin sections revealed peripheral nerve fascicles with severe loss of myelinated fibers (about 95% compared to healthy controls) and only few remaining myelinated fibers (Figures 1C and 1D). Single fibers with acute myelin degradation could be observed. Electron microscopy confirmed the severe axonal neuropathy with few remaining myelinated fibers with signs of axonal atrophy, few myelin debris and denervated Schwann cell bands (Figures 1E and 1F). Additionally, a severe loss of unmyelinated fibers could be detected. Single amyloid deposits were demonstrated by ultrastructural analysis. There were no signs of axonal regeneration.

In the genetic analyses of the *TTR* gene, we found a heterozygous missense mutation: c.221A>G (GAG>GGG; p.Glu74Gly). The four coding exons of *TTR*, corresponding to coding cDNA nucleotides c.1 to c.444, including flanking sequences, were amplified by PCR and screened for mutations by direct sequencing (reference sequence: TTR ENSG00000118271 with transcript ENST00000237014). Mutation designation according to standard naming conventions of the Human Genome Variation Society [9], which uses numbering beginning at the Met initiation codon, includes a 20–amino acid signal sequence and may be different from that reported in the literature. The alternative mutation designation for the mutation detected in our patient is p.Glu54Gly.

Treatment with tafamidis was initiated after TTR-FAP was diagnosed [10], but our patient’s neuropathy was aggravated nonetheless. An orthotopic liver transplant was performed 10 months later, but the patient died as a result of intra-operative complications. Three of her four brothers and three sisters have since presented to our outpatient clinic. A 36-year-old brother tested positive for amyloid in a subcutaneous fat aspiration test. Further family members in Turkey have been evaluated for the mutation. One cousin was found to be a mutation carrier; unfortunately, we have no further information on his health condition.

## Discussion

Hereditary amyloidoses with a predominant involvement of the peripheral nervous system are rare and often difficult to diagnose, especially when other family members are not affected. The patients are often misdiagnosed, leading to a delay in the initiation of therapy. In our patient, a rapidly progressive neuropathy with initial symptoms of small fiber involvement was the leading symptom. The major findings were the nerve biopsy with endoneural amyloid deposits and a severe loss of myelinated and unmyelinated fibers in the absence of signs of regeneration.

Some rare mutations of familial amyloid neuropathies are associated with an early onset and a rapid disease progression. Patients with the rare mutation in p.Glu74Gly (pGlu54Gly) usually present with a positive family history and have severe autonomic disturbance causing gastrointestinal symptoms, such as vomiting, constipation and diarrhea, of many years’ duration [6, 11, 12]. Moreover, blindness is an early phenomenon in patients with a mutation in Glu54Gly [11, 12]. Our patient did not have any of these symptoms. Although she did not complain about visual disturbances, opacity of the vitreous body with signs of visual loss could be detected.

In this report, we present the case of a 35-year-old woman of Turkish origin who had TTR-FAP with a rare mutation with a phenotype different from those in other cases reported in the literature so far. First, our patient’s family history was unremarkable. Second, she reported small fiber involvement in the sense of a small fiber neuropathy without typical autonomic signs. Early involvement of small nerve fibers (Aδ and C fibers) in TTR-FAP patients is well described in the literature [3, 7, 13–15]. However, the typical autonomic disturbances described in patients with a mutation in Glu54Gly, such as gastrointestinal symptoms, were completely absent in our patient. Third, no visual disturbances were present; however, upon examination, opacity of the vitreous body could be detected. Therefore, the severe polyneuropathy was the only symptom of this very rare mutation in our patient.

## Conclusion

An amyloid neuropathy should be considered in patients with progressive neuropathies, even if the neuropathy is the only complaint and the patient’s family history is negative.

## Consent

Written informed consent was obtained for publication of this case report from the three tested brothers of our deceased patient. Information regarding the other sibling was obtained through the three brothers and the patient. A copy of the written consent is available for review by the Editor-in-Chief of this journal.

## Abbreviations

TTR-FAP: Transthyretin-associated familial amyloidotic polyneuropathy.
